# Silver Nanoparticles (AgNPs) as Enhancers of Everolimus and Radiotherapy Sensitivity on Clear Cell Renal Cell Carcinoma

**DOI:** 10.3390/antiox12122051

**Published:** 2023-11-28

**Authors:** Mariana Morais, Vera Machado, Patrícia Figueiredo, Francisca Dias, Rogéria Craveiro, Joana Lencart, Carlos Palmeira, Kirsi S. Mikkonen, Ana Luísa Teixeira, Rui Medeiros

**Affiliations:** 1 Molecular Oncology and Viral Pathology Group, Research Center of IPO Porto (CI-IPOP)/RISE@CI-IPOP (Health Research Network), Portuguese Oncology Institute of Porto (IPO-Porto)/Porto Comprehensive Cancer Center (Porto.CCC), Research Center-LAB2, E Bdg 1st Floor, Rua Dr António Bernardino de Almeida, 4200-072 Porto, Portugal; mariana.gomes.morais@ipoporto.min-saude.pt (M.M.); verapm28@gmail.com (V.M.); francisca.carvalho.dias@ipoporto.min-saude.pt (F.D.); ruimedei@ipoporto.min-saude.pt (R.M.); 2 ICBAS, Abel Salazar Institute for the Biomedical Sciences, University of Porto, Rua Jorge Viterbo Ferreira 228, 4050-513 Porto, Portugal; 3 Department of Food and Nutrition, Faculty of Agriculture and Forestry, University of Helsinki, FI-00014 Helsinki, Finland; patricia.figueiredo@helsinki.fi (P.F.); kirsi.s.mikkonen@helsinki.fi (K.S.M.); 4 Radiobiology and Radiological Protection Group, Research Center of IPO Porto (CI-IPOP)/RISE@CI-IPOP (Health Research Network), Portuguese Oncology Institute of Porto (IPO-Porto)/Porto Comprehensive Cancer Center (Porto.CCC), Rua Dr António Bernardino de Almeida, 4200-072 Porto, Portugal; rogeriapereira@ipoporto.min-saude.pt (R.C.); i1934@ipoporto.min-saude.pt (J.L.); 5 Department of Medical Physics, Portuguese Oncology Institute of Porto (IPO-Porto), Rua Dr António Bernardino de Almeida, 4200-072 Porto, Portugal; 6 Department of Immunology, Portuguese Oncology Institute of Porto (IPO-Porto), Rua Dr António Bernardino de Almeida, 4200-072 Porto, Portugal; carlospalmeira@ipoporto.min-saude.pt; 7 Experimental Pathology and Therapeutics Group, Research Center of IPO Porto (CI-IPOP)/RISE@CI-IPOP (Health Research Network), Portuguese Oncology Institute of Porto (IPO-Porto)/Porto Comprehensive Cancer Center (Porto.CCC), Research Center-LAB2, E Bdg 1st floor, Rua Dr António Bernardino de Almeida, 4200-072 Porto, Portugal; 8 Helsinki Institute of Sustainability Science (HELSUS), University of Helsinki, FI-00014 Helsinki, Finland; 9 Biomedical Reasearch Center (CEBIMED), Faculty of Health Sciences, Fernando Pessoa University (UFP), Praça 9 de Abril 349, 4249-004 Porto, Portugal; 10 Research Department, LPCC-Portuguese League Against Cancer (NRNorte), 4200-172 Porto, Portugal; 11 Faculty of Medicine, University of Porto (FMUP), Alameda Prof. Hernâni Monteiro, 4200-319 Porto, Portugal

**Keywords:** nanotechnology, silver nanoparticles, cancer, renal cell carcinoma, radiotherapy, targeted therapies, reactive oxygen species

## Abstract

Nanomedicine’s advent has promised to revolutionize different biomedical fields, including oncology. Silver Nanoparticles (AgNPs) showed promising results in different tumor models. Clear cell Renal Cell Carcinoma (ccRCC) is especially challenging due to its late diagnosis, poor prognosis and treatment resistance. Therefore, defining new therapeutic targets and regimens could improve patient management. This study intends to evaluate AgNPs’ effect in ccRCC cells and explore their potential combinatory effect with Everolimus and Radiotherapy. AgNPs were synthesized, and their effect was evaluated regarding their entering pathway, cellular proliferation capacity, ROS production, mitochondrial membrane depolarization, cell cycle analysis and apoptosis assessment. AgNPs were combined with Everolimus or used to sensitize cells to radiotherapy. AgNPs are cytotoxic to 786-O cells, a ccRCC cell line, entering through endocytosis, increasing ROS, depolarizing mitochondrial membrane, and blocking the cell cycle, leading to a reduction of proliferation capacity and apoptosis. Combined with Everolimus, AgNPs reduce cell viability and inhibit proliferation capacity. Moreover, 786-O is intrinsically resistant to radiation, but after AgNPs’ administration, radiation induces cytotoxicity through mitochondrial membrane depolarization and S phase blockage. These results demonstrate AgNPs’ cytotoxic potential against ccRCC and seem promising regarding the combination with Everolimus and sensitization to radiotherapy, which can, in the future, benefit ccRCC patients’ management.

## 1. Introduction

Renal Cell Carcinoma (RCC) is the 12th most common cancer worldwide, accounting for 2.4% of all neoplasias in adults. By 2020, according to GLOBOCAN, there were 431,000 new cases diagnosed and about 180,000 deaths, representing 1.8% of all cancer deaths worldwide. Moreover, incidence rates are projected to increase in the future decades [1].

RCC is a highly heterogeneous disease with different genetic, molecular, and phenotypic characteristics. Clear Cell RCC (ccRCC) is the most common and the one with the worst prognosis, comprising 75% of all RCC cases [2]. It is originated from tubular epithelial tissue, often metastasizing to the lungs or bone and in 90% of the cases, is associated with *Von Hippel-Lindau* (*VHL*) gene mutations, which leads to a constitutive expression of *hypoxia-inducible factor (HIF-1,2*), leading to an increased expression genes related to angiogenesis, growth factors and glucose metabolism [3].

In the last years, because of the increased use of computed tomography and magnetic resonance imaging, there has been an increase in the detection of early RCC cases [4]. In these cases, nephrectomy is the recommended treatment, with a high survival rate. However, RCC patients with metastases show a 5-year survival rate of only 12% [5].

Due to its hypoxic nature, RCC is commonly resistant to both chemo- and radiotherapy. Thus, the management of metastatic disease highly depends on targeted therapies, such as anti-vascular endothelial growth factor (VEGF) antibodies, VEGF receptor tyrosine kinase inhibitors, mammalian target of rapamycin (mTOR) pathways inhibitors and immune checkpoint inhibitors [6]. Nevertheless, patients end up developing resistance to these agents, and a quarter of patients do not seem to show any clinical benefit. In fact, patients develop resistance in a median of 5–11 months, making the development of novel therapeutic agents an urgent need [7].

Thus, we hypothesized that nanomedicine has come up as an attractive approach towards biotechnology and biomedicine in different fields, such as oncology. In fact, several nanoparticles (NPs) have already been approved for clinical use by both the Food and Drug Administration and the European Medicines Agency [8]. Among the different NPs are silver Nanoparticles (AgNPs), which show specific physical and chemical characteristics that have been shown promising as strategies to improve treatment and overcome therapy resistance in different tumor models [9].

In fact, we have previously synthesized AgNPs that showed a promising cytotoxicity against Prostate Cancer cells that are resistant to hormone treatments, which is one of the main options in the management of patients with advanced disease [10]. These AgNPs were previously characterized through ultra-violet (UV) visible spectroscopy, dynamic light scattering (DLS), transmission electron microscopy (TEM), Fourier transform infrared spectroscopy (FTIR) and their stability after incubation with cell culture medium regarding changes in size, PDI and ζ-potential [10]. These AgNPs showed a peak Ultraviolet visible (UV-Vis) absorption at around 412 nm, an average size of 61 ± 10 nm, a spherical shape, a moderate monodisperse nature and a highly negative surface charge (−59 ± 6 mV), mostly due to tannic acid and sodium citrate present in their surface [10]. Moreover, after contact with the cell culture medium, they remained stable [10]. They entered cells through endocytosis and depolarized the mitochondrial membrane, leading to the production of reactive oxygen species (ROS), which, in turn, disrupted cells’ cell cycle, blocked their proliferation and ultimately led to apoptosis [10].

Therefore, and since the state of pseudo-hypoxia of ccRCC cells is somehow responsible for their resistance to radiotherapy and mTOR inhibitors, we hypothesized that these AgNPs may show cytotoxic effect regarding ccRCC cells and sensitize them to radiotherapy [11,12]. Thus, the aim of this study was to evaluate AgNPs’ cytotoxic effect in ccRCC cells, their combined effect with the mTOR inhibitor Everolimus and their potential as radiation sensitizers.

## 2. Materials and Methods

### 2.1. Synthesis of AgNPs

AgNPs were synthesized and characterized as described by Morais et al. [10]. Briefly, an aqueous solution of 100 mL containing sodium citrate (5 mM) and tannic acid (5 mM) was mixed and heated at 100 °C, for 15 min under vigorous stirring. After starting to boil, 1 mL of AgNO_3_ (25 mM) was added to this solution for 5 min. AgNPs were purified by centrifugation at 20,000× *g* for 15 min and redispersed in MilliQ (MQ) water.

### 2.2. Cell Culture and Treatment Conditions

A renal cell adenocarcinoma cell line, 786-O (ATCC^®^ CRL-1932^TM^ RRID: CVCL_1051), was kindly granted by Professor Carmen Jerónimo (IPO-Porto Research Center- Portugal). RCC-FG2 (CLS catalog number 300249) is a metastatic ccRCC cell line kindly provided by Dr. Klaas Kok from Groningen University (The Netherlands). Caki-1 is a metastatic ccRCC cell line (ATCC^®^ HTB-46^TM^ RRID: CVCL_0234) obtained from ATCC. The hepG-2 cell line is a hepatoma cell line and was kindly granted by the Basic and Clinical Research on Iron Biology Group from I3S. 786-O, RCC-FG-2 and Caki-1 cells were kept in RPMI-1640 medium, and HepG-2 cells were kept in DMEM (Sigma Aldrich^®^, St. Louis, Missouri, EUA) medium, all supplemented with 10% fetal bovine serum (FBS) and 1% penicillin-streptomycin in a humidified incubator at 37 °C, 5% CO_2_ and 95% humidity. Cells were trypsinized at 80–90% confluence with trypsin-EDTA (1X) (Gibco^®^, Gaithersburg, MD, USA) and counted using trypan blue solution (VWR^TM^) and an automatic cell counter (EVE^TM^—NanoEntek, Hwaseong, Gyeonggi-do Province, Republic of Korea). All cells were weekly tested for mycoplasma presence and were found to be free from contamination.

### 2.3. AgNPs IC_25_ and IC_50_ Determination by Resazurin Assay

All cell lines were plated in 96-multi-well plates at a concentration of 2 × 10^5^ cells/mL, and AgNPs were administrated at a concentration range of 500–3000 µg/mL for 24 h. Resazurin Sodium Salt (ACROS Organic™—Fisher Scientific^®^, Hampton, New Hampshire, USA) was used to assess cells’ viability. Two biological replicates and six technical replicates were performed for all conditions. IC_50_ was obtained through The Quest Graph™ IC_50_ Calculator, with a four-parameter logistic regression model, and IC_25_ was determined through the following equation: IC_25_ $={\left(\frac{25}{100-25}\right)}^{\frac{1}{HillSlope}}\times$ IC_50_. 786-O was the cell line in which AgNPs showed the most promising effect and was the one chosen for the following essays, which were performed in the IC_25_ and IC_50_.

### 2.4. Determination of Cellular Localization of AgNPs by Transmission Electron Microscopy (TEM)

786-O cells were plated in 6-multi-well plates at a 2 × 10^5^ cells/mL concentration, and the AgNPs were administrated at IC_25_ for 24 h. After, cells were trypsinized with trypsin-EDTA, collected, fixed, and further processed. TEM images were collected with Transmission Electron Microscope Jeol JEM 140 by the Histology and Electron Microscopy platform from I3S Porto.

### 2.5. Cell Proliferation Assay—BrdU

To assess the proliferation capacity of cells after AgNPs treatment, a BrdU assay was performed, as previously described, using the Cell Proliferation ELISA, BrdU kit, following manufacturer instructions (Roche Diagnostics, Mannheim, Germany) [10]. Results were expressed as a percentage of control (100%). Five technical replicates were performed for each condition.

### 2.6. Cell Cycle Analysis

Cell cycle was assessed by Propidium Iodide (PI) and flow cytometry, as previously described [10]. Briefly, cells were plated in 6-well plates at a concentration of 2 × 10^5^ cells/mL and incubated with IC_25_ and IC_50_ of AgNPs for 24 h, with three replicates each. The culture medium was collected, cells were detached, and the pellet was washed and fixed. Fixed cells were then stained with a solution of Triton X-100 (1.1%, *v*/*v*), PI (50 μg/mL) and RNase A (100 μg/mL) in PBS. The cell cycle was analyzed with a flow cytometer (Cytomics FC500, Beckman Coulter, Brea, CA, USA). Cells’ percentage in the sub-G1 phase was calculated from the total of cells, and the percentage of cells in the remaining phases was calculated from all cells except sub-G1 cells.

### 2.7. Cell Apoptosis Assay

FITC annexin V and PI. Apoptosis was assessed by the Annexin V-FITC Apoptosis Detection kit (Abcam, Cambridge, UK) according to the manufacturer’s instructions, as previously described [10]. Cells were cultured in 6-well plates (2 wells per condition) at a concentration of 2 × 10^5^ cells/mL and incubated with the IC_25_ and IC_50_ of AgNPs for 24 h. Then, cells were detached and stained using recombinant annexin V conjugated to fluorescein (FITC annexin V) and red-fluorescent PI. Data analysis was performed through CXP Software 2003 in an FC500 Beckman Coulter flow cytometer.

### 2.8. Detection of Intracellular Reactive Oxygen Species

DCFH_2_-DA assay. Intracellular ROS production was evaluated through fluorescence of DCF measurement. Cells were plated in 96-well plates at a concentration of 2 × 10^5^ cells/mL, and AgNPs were administrated at IC_25_ and IC_50_ for 24 h. Three hours before the end of treatment, 150 µM tert-butyl hydrogen peroxide (t-bhp) (Sigma-Aldrich^®^, St. Louis, MO, USA) was added to naïve cells as positive control. T-bhp mimics ROS activity oxidizing DCFDA to fluorescent DCF. Then, 10 µM DCFH_2_-DA (Merck Millipore^®^, Burlington, MA, USA) was added, and live cells were imaged as previously described [10].

### 2.9. Mitochondrial Membrane Potential Assay (TMRE)

To evaluate mitochondrial membrane potential depolarization, tetramethylrhodamine, ethyl ester (TMRE) (Abcam, Cambridge, UK) was used according to the manufacturer’s instructions. Cells were plated in 96-well plates with clear flat bottom and black sides at a concentration of 2 × 10^5^ cells/mL and the IC_25_ and IC_50_ of AgNPs was administrated for 24 h. Carbonyl cyanide-p-trifluoromethoxyphenylhydrazone (FCCP) was used as a positive control at the concentration of 100 µM for 10 min. Then, 500 nM of TMRE was administrated for 20 min at 37 °C, 5% CO_2_. Finally, cells were washed with 100 µL of PBS containing 0.2% bovine serum albumin (BSA). A volume of 100 µL of PBS/0.2% BSA was added to each well, and fluorescence was measured in a microplate fluorescence reader (Flx 800, Biotek, Winooski, VT, USA) with excitation/emission: 549/575 nm. Additionally, live cells were imaged with a filter set appropriate for Tetramethylrhodamine (TRITC) with a fluorescence microscope OLYMPUS IX51 (Tokyo, Japan). Two biological replicates and five technical replicates were performed for each condition.

### 2.10. HepG-2 Toxicity Evaluation—Resazurin Assay

HepG-2 cells were plated in 96-well plates at 2.0 × 10^5^ cells/mL in a final volume of 100 µL. Twenty-four hours later, AgNPs were administrated at IC_50_ of 786-O to assess biocompatibility and resazurin sodium salt was used to assess cells’ viability.

### 2.11. Everolimus and AgNPs Combined Effect Evaluation

To evaluate the combined effect of Everolimus (an mTOR inhibitor used in ccRCC treatment) and AgNPs, a 13.5 μM concentration of Everolimus was selected based on an intermediate concentration of the doses’ range used to induce resistance in 786-O in a previous group work (5–20 μM) [11]. Cells were administrated with 13.5 µM Everolimus alone and in combination with IC_25_ and IC_50_ of AgNPs. The combination of the lowest dose of both Everolimus and AgNPs was chosen for the following assays.

### 2.12. Irradiation

Irradiation was performed at room temperature using a 6 MV photon beam generated in a Varian TrueBeam^®^ accelerator, with a dose rate of 6 Gy per minute. The irradiation field was such that uniform doses of 2 Gy, 4 Gy, 8 Gy, 20 Gy and 80 Gy were delivered to the cells. After 1 h, their metabolic and proliferation capacity, cell cycle and apoptosis status, mitochondrial membrane potential and production of ROS were assessed as described above. 8 Gy was the chosen dose for the following assays. To evaluate AgNPs’ potential as sensitizing agents for radiation, 786-O cells were plated at a concentration of 2 × 10^5^ cells/mL and AgNPs were administrated at IC_25_ concentration, for 24 h. After, dead cells were removed by washing with a culture medium and the remaining live cells were submitted to radiation (8 Gy). After 1 h, the assays previously described were performed.

### 2.13. Statistical Analysis

SPSS28 software (release 28, SPSS Inc., Chicago, IL, USA) was used to analyze results through the Sample *t*-test, Mann–Whitney and Kruskal–Wallis tests according to the normality state of the data. *p*-values < 0.05 were considered statistically significant.

## 3. Results

### 3.1. AgNPs’ Cytotoxic Potential Screening in RCC Cell Lines

The AgNPs used in this study were characterized in our previous work [10]. Briefly, they show an average size of 61 ± 10 nm, moderate monodispersity (PDI values lower than 0.25) and a highly negative surface charge (−59 ± 6 mV). They showed a maximum UV-Vis absorption at circa 412 nm, present a roughly round shape, and are known to remain stable and disperse for over a month in water. Moreover, they were proven only to suffer a slight increase in size and to remain monodisperse in a cell culture medium.

**Figure 1:**
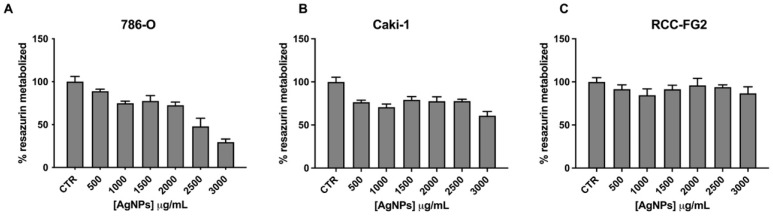
Cell viability assessment through Resazurin Assay, upon AgNPs incubation for 24 h in a 786-O cell line (**A**), Caki-1 cell line (**B**) and RCC-FG2 cell line (**C**). Results are expressed as a percentage of control (untreated cells), as mean ± SEM.

To evaluate their cytotoxic effect in ccRCC cells, the viability of three ccRCC cell lines was screened with AgNP doses ranging from 500 to 3000 µg/mL for 24 h (Figure 1). While one can see a dose-dependent effect of AgNPs in 786-O cell line (Figure 1A), the same does not happen regarding both Caki-1 and RCC-FG2, where the effect is lower and does not increase with AgNPs’ dose (Figure 1B,C). Thus, AgNPs show a higher cytotoxic potential against the 786-O cell line, and this was the cell line chosen for the remaining studies.

### 3.2. The Phenotypic Effect of AgNPs in 786-O Cell Line

To go further with the study of the phenotypic effect of AgNPs in the 786-O cell line, we first determined the IC_25_ and IC_50_ (Table 1).

**Figure 2:**
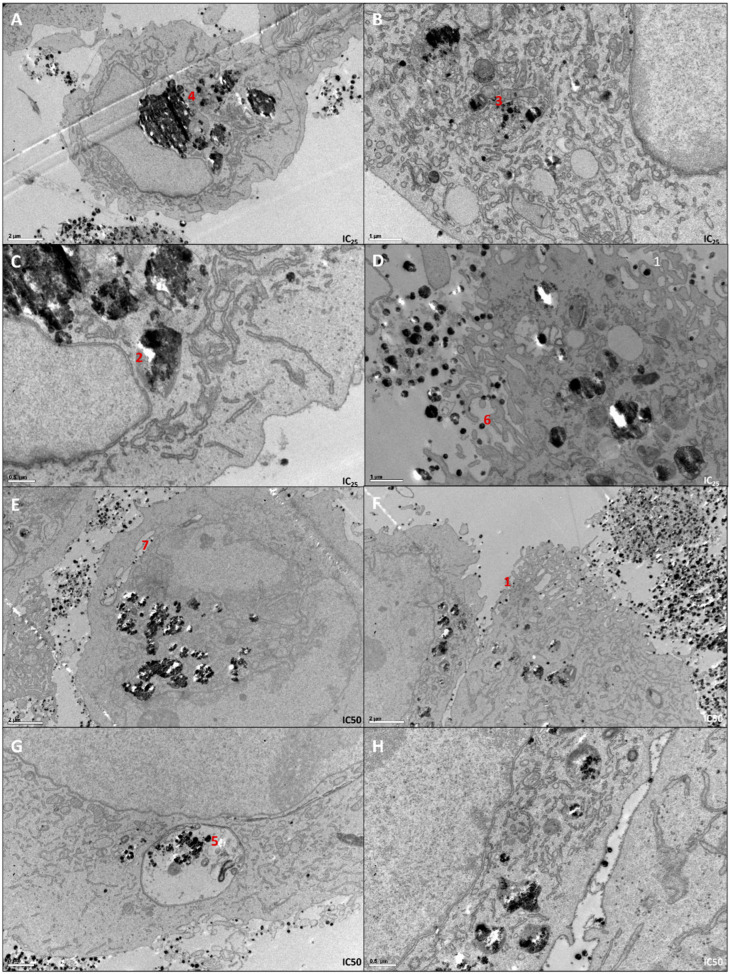
TEM images of 786-O cells treated with IC_25_ (**A**–**D**) and IC_50_ (**E**–**H**) of AgNPs. From each sample, pictures were taken with different ampliations. Thus, scale bars in (**A**,**E**,**F**) are 2 µm, in (**B**,**D**,**G**) are 1 µm and in (**C**,**H**) are 0.5 µm. 1—Cell invaginations capturing AgNPs; 2—Nanoparticles localized near the nucleus; 3—Nanoparticles localized near mitochondria; 4—Nanoparticles localized near Golgi complex; 5—Nanoparticles trapped inside lysosomes; 6—Apoptotic features of 786-O cells; 7—Nanoparticles monodisperse nature.

Using these two concentrations, a TEM analysis was performed in order to evaluate the internalization process and AgNPs’ location inside the cell (Figure 2). Through TEM analysis, it is possible to observe that cells seem to form invaginations to internalize the AgNPs (Figure 2F1). Inside the cell, AgNPs are located near the nucleus (Figure 2C2), mitochondria (Figure 2B3) and the Golgi complex (Figure 2A4), being integrated inside vacuoles resembling lysosomes (Figure 2G5). It is important to notice that cells display apoptotic features (Figure 2D6) and AgNPs keep their monodisperse nature inside the cell (Figure 2E7).

**Figure 3:**
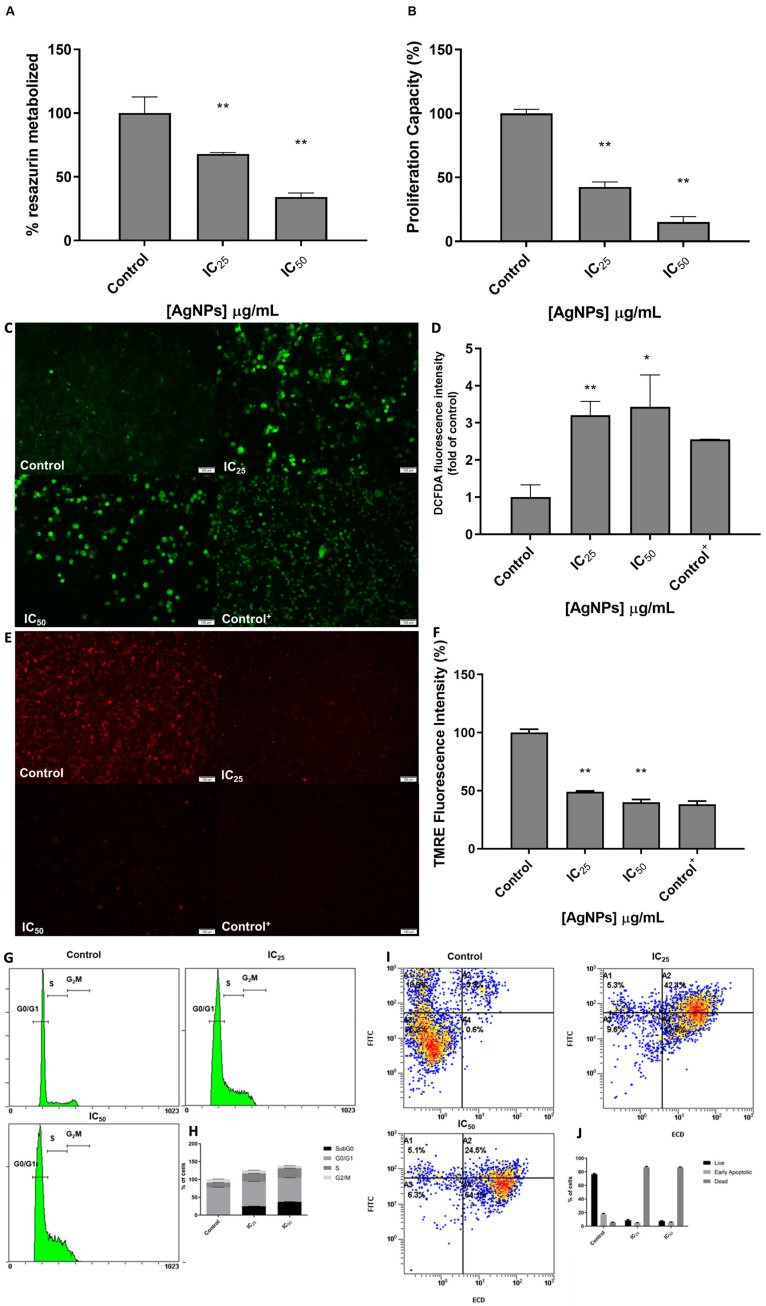
Evaluation of the phenotypic effect of 24 h treatment with IC_25_ and IC_50_ AgNPs in 786-O cells. (**A**) Cell viability, assessment by Resazurin Assay. Results are expressed as percentage of control (untreated cells), as mean ± SEM. Statistical analysis was performed in comparison to control; ** *p* < 0.001. (**B**) Proliferation capacity assessment by BrDu Assay. Results are expressed as a percentage of control (untreated cells), as mean ± SEM. Statistical analysis was performed in comparison to control; ** *p* < 0.001. (**C**) Evaluation of ROS production by DCFH2-DA assay; Representative Images (10×) taken using an Olympus IX51 microscope, scale bars represent 100 μM. (**D**) Graph representing the intensity of DCFH2-DA fluorescence signal. T-bhp represents positive control. Results expressed as fold of control (untreated cells), as mean ± SEM Statistical analysis was performed in comparison to control; * *p* < 0.05; ** *p* < 0.001. (**E**) Evaluation of mitochondria membrane depolarization, by TMRE Assay; Representative Images (10×) taken using an Olympus IX51 microscope, scale bars represent 100 μM. (**F**) Graph representing the intensity of TMRE fluorescence signal. FCCP represents positive control. Results are expressed as a percentage of control (untreated cells), as mean ± SEM. Statistical analysis was performed in comparison to control; ** *p* < 0.001. (**G**) Representative DNA histogram of cell cycle analysis evaluated through the PI stain and flow cytometry. (**H**) Descriptive analysis of the number of cells in each phase of the cell cycle. Data are expressed as mean ± SEM. (**I**) Representative flow cytometry images of annexin (FITC) and PI (ECD) staining regarding apoptosis assessment. (**J**) Descriptive analysis of the number of live, early apoptotic and dead cells; Data are expressed as mean ± SEM.

Cells were then analyzed regarding their viability and proliferation capacity when administrated with IC_25_ and IC_50_. Their viability, assessed through the % of resazurin metabolized, was significantly lower after administration of IC_25_ of AgNPs (*p* < 0.001) and IC_50_ (*p* < 0.001) (Figure 3A). The same is observed regarding proliferation capacity (Figure 3B). After administration of IC_25_ of AgNPs, cells’ proliferation is inhibited to 42.58% (*p* < 0.001) and after administration of IC_50_ (*p* < 0.001), cells’ proliferation is inhibited to 15.19% (*p* < 0.001).

To evaluate AgNPs’ effect on mitochondria, cells were assessed regarding their production of ROS and the polarization state of the mitochondrial membrane (Figure 3C,E). After administration of AgNPs IC_25_ and IC_50_, there was a 3.21 and 3.43 fold-increase in ROS levels (*p* < 0.001, *p* < 0.001), respectively (Figure 3D). In fact, after administration of IC_25_ of AgNPs, we observed that cells were depolarized to 49.01% (*p* < 0.001), and after administration of IC_50_ of AgNPs, cells were depolarized to 39.9% (*p* < 0.001) (Figure 3F).

Furthermore, the effect of AgNPs on cell cycle and apoptosis of 786-O cells was also evaluated (Figure 3G,I). Both IC_25_ and IC_50_ of AgNPs increased the number of cells in the S phase (22.30% vs. 13.28% and 25.49% vs. 13.28%, respectively) and decreased the number of cells in G2/M (8.84% vs. 9.88% and 8.41% vs. 9.88%, respectively), as well as increased the number of cells in subG0 (24.87% vs. 0.74% and 24.27% vs. 0.74%, respectively) (Figure 3H). Regarding their apoptotic state, one can see that most cells were positive for both Annexin and PI, validating their apoptotic status after administration of these particles (Figure 3J).

### 3.3. AgNPs Biocompatibility in HepG-2 Cell Line

**Figure 4:**
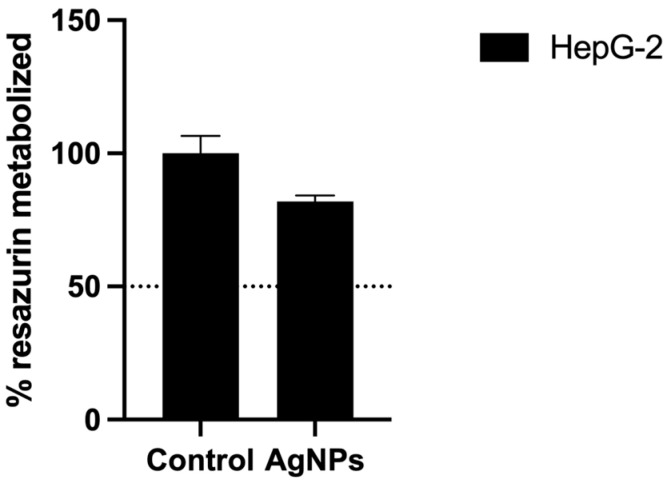
Evaluation of cell viability in the HepG-2 cell line by Resazurin assay upon treatment with the determined IC_50_ of AgNPs of 786-O cells. Results are expressed as a percentage of control (untreated cells), as mean ± SEM.

As a preliminary screening approach to the biocompatibility of AgNPs, the HepG-2 cell line was used to evaluate in vitro hepatotoxicity. The previously determined IC_50_ of 786-O cells was administrated to HepG-2 cells. One can observe that, in the HepG-2 cells, AgNPs at a concentration of 2629.63 µg/mL reduced cell viability to 81.97% against the 50% observed in 786-O cells (Figure 4).

### 3.4. AgNPs and Everolimus Combined Effect in 786-O Cell Line

**Figure 5:**
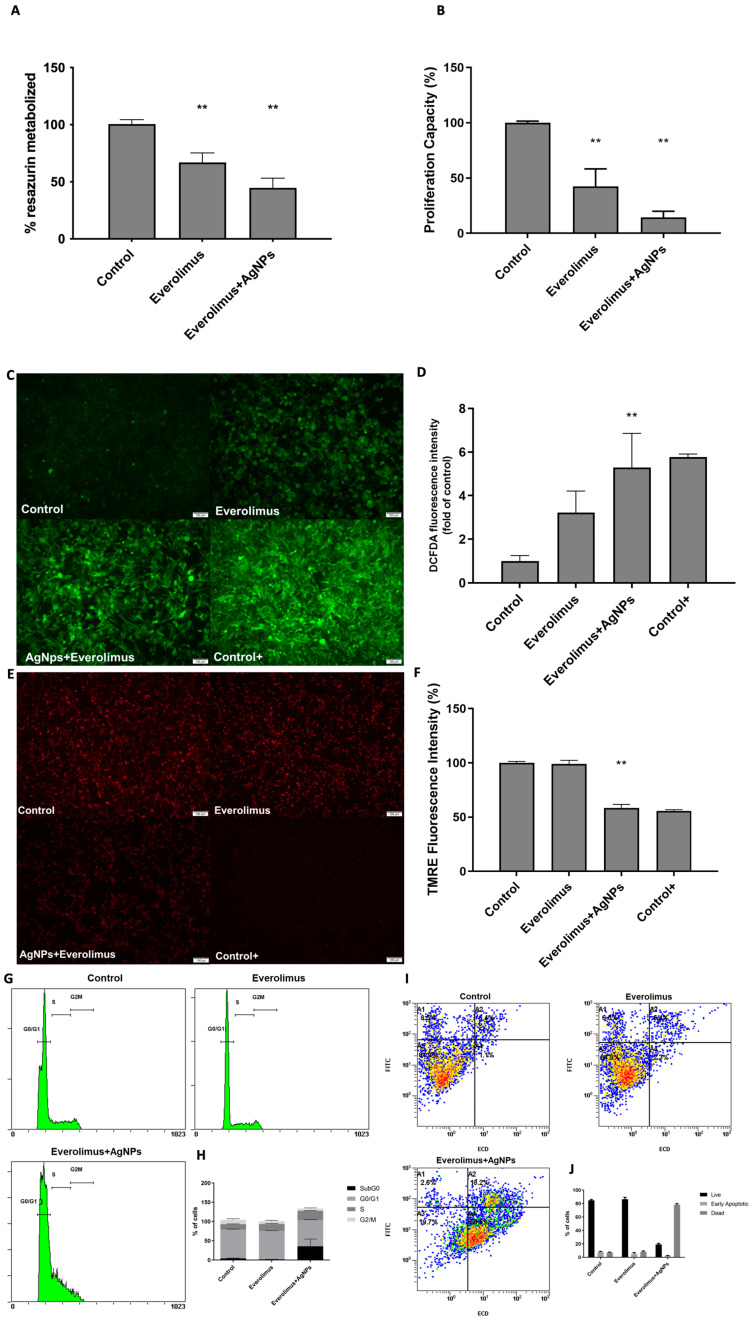
Evaluation of the phenotypic effect of the combined treatment of AgNPs and Everolimus in 786-O cells. (**A**) Cell viability assessment by Resazurin Assay. Results are expressed as a percentage of control (untreated cells), as mean ± SEM. Statistical analysis was performed in comparison to control; ** *p* < 0.001. (**B**) Proliferation capacity assessment of cells treated with 13.5 µM alone or in combination with IC_25_ of AgNPs by BrDu Assay. Results are expressed as a percentage of control (untreated cells), as mean ± SEM. Statistical analysis was performed in comparison to control; ** *p* < 0.001. (**C**) Evaluation of ROS production of cells treated with 13.5 µM alone or in combination with IC_25_ of AgNPs, by DCFH2-DA assay; Representative Images (10×) taken using an Olympus IX51 microscope. (**D**) Graphic representing the intensity of DCFH2-DA fluorescence signal. T-bhp represents positive control. Results are expressed as fold of control (untreated cells), as mean ± SEM Statistical analysis was performed in comparison to control; ** *p* < 0.001. (**E**) Evaluation of mitochondria membrane depolarization of cells treated with 13.5 µM alone or in combination with IC_25_ of AgNPs, by TMRE Assay; Representative Images (10×) taken using an Olympus IX51 microscope. (**F**) Graphic representing the intensity of the TMRE fluorescence signal. FCCP represents positive control. Results are expressed as a percentage of control (untreated cells), as mean ± SEM. Statistical analysis was performed in comparison to control; ** *p* < 0.001 (**G**) Representative DNA histogram of cell cycle analysis of cells treated with 13.5 µM alone or in combination with IC_25_ of AgNPs, evaluated through PI stain and flow cytometry. (**H**) Descriptive analysis of the number of cells in each phase of the cell cycle. Data are expressed as mean ± SEM. (**I**) Representative flow cytometry images of annexin (FITC) and PI (ECD) staining regarding apoptosis assessment of cells treated with 13.5 µM alone or in combination with IC_25_ of AgNPs; the different colors in the picture represent the crescent density of cells (blue-less density; red-more density). (**J**) Descriptive analysis of the number of live, early apoptotic and dead cells; Data are expressed as mean ± SEM.

To understand the potential of AgNPs as therapeutic agents that potentiate the effect of current standard treatment approved for ccRCC, we studied their combined effect with Everolimus, an mTOR pathway inhibitor used as a second-line and third-line treatment in ccRCC [12]. After administration of Everolimus at 13.5 µM, there was a significative decrease in 786-O viability to 66.88% (*p* < 0.001). When administrated in combination with IC_25_ AgNPs, there was a significative decrease in 786-O viability to 44.64% (*p* < 0.001) (Figure 5A).

Analyzing the proliferation capacity of 786-O cells, it is possible to observe that administration of Everolimus 13.5 µM led to a reduction of proliferation capacity to 42.44% (*p* < 0.001) and the combination of Everolimus 13.5 µM with IC_25_ AgNPs led to a higher reduction of proliferation capacity to 14.45% (*p* < 0.001) (Figure 5B). Regarding mitochondrial function, cells were assessed regarding their production of ROS and the polarization state of the mitochondrial membrane (Figure 5C,E). Interestingly, there are no significant differences after the administration of Everolimus 13.5 µM (*p* = 0.083). However, the combination of Everolimus 13.5 µM and AgNPs IC_25_ led to a 5.28-fold increase in ROS (*p* = 0.022), a higher ROS production considering the single effect of AgNPs mentioned above (Figure 5D).

The same was observed regarding mitochondrial membrane depolarization. There were no significative differences after administration of Everolimus 13.5 µM (*p* = 0.693). However, the combination of Everolimus 13.5 µM and AgNPs IC_25_ led to a decrease in polarized mitochondrial membrane to 59.40 (*p* < 0.001) (Figure 4F). Lastly, we analyzed the phenotypic effect of this combination on cell cycle status and apoptosis (Figure 5G,I). We found that the administration of Everolimus 13.5 µM is associated with a slight increase in the number of cells in the S phase (13.93% vs. 17.86%) followed by a decrease in the number of cells in the G2/M phase (11.43% vs. 7.23%). Nevertheless, the combination of Everolimus 13.5 µM with AgNPs IC_25_ leads to a sharper increase in the number of cells in the S phase (31.93% vs. 24.42%) and a consequent decrease in the number of cells in the G2/M phase (11.43% vs. 5.85%), as well as induce an increase in cells in subG0 (4.31% vs. 35.72%) (Figure 4H). Regarding their apoptotic state, one can see that cells were marked positive for both Annexin and PI (thereby, being apoptotic) only when administrated with the combination of Everolimus 13.5 µM with AgNPs IC_25_ (Figure 5J).

### 3.5. AgNPs Effect as Sensitizers to Radiation in 786-O Cell Line

**Figure 6:**
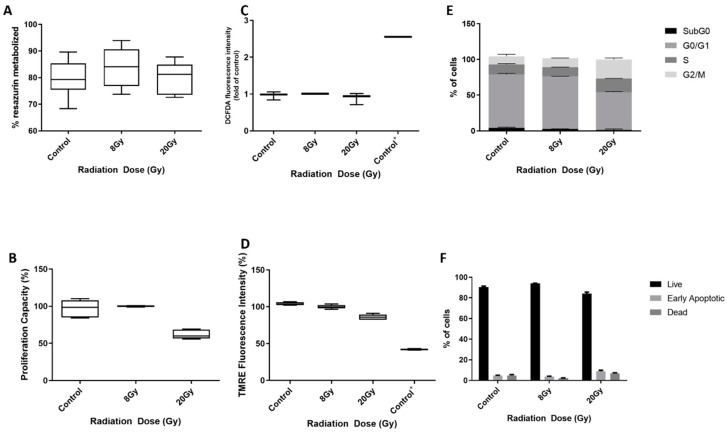
Effect of radiation (8 Gy and 20 Gy) in 786-O cells. Cells were exposed to radiation, and the effect was evaluated 1 h after the stimulus. (**A**) Cell viability assessment by Resazurin Assay. Results are expressed as a percentage of control (untreated cells), as mean ± SEM. (**B**) Proliferation capacity assessment by BrDu Assay. Results are expressed as a percentage of control (untreated cells), as mean ± SEM. (**C**) ROS production assessment through DCFH2-DA. T-bhp represents positive control. Results are expressed as fold of control (untreated cells), as mean ± SEM (**D**) Evaluation of mitochondria membrane depolarization, by TMRE Assay; FCCP is used as positive control. Results are expressed as a percentage of control (untreated cells), as mean ± SEM. (**E**) Descriptive analysis of the number of cells in each phase of the cell cycle. Data are expressed as mean ± SEM (**F**) Descriptive analysis of the number of live, early apoptotic and dead cells; Data are expressed as mean ± SEM.

Since primary resistance to radiotherapy is one of the many challenges of ccRCC treatment, we studied AgNPs’ capacity to sensitize these tumors to this therapy. Firstly, we assessed the effect of radiation alone on 786-O cells (Figure 6). To do so, we exposed cells to two different doses of radiation (8 Gy and 20 Gy) and assessed their viability 1 and 24 h after exposure. We assessed cells’ viability (Figure 6A), proliferation capacity (Figure 5B), ROS production (Figure 5C), mitochondrial membrane polarization (Figure 5D), cell cycle status (Figure 5E) and apoptosis (Figure 5F) 1 h after exposure. As observed, neither 8 Gy nor 20 Gy of radiation were able to produce any damage in the different tested parameters.

**Figure 7:**
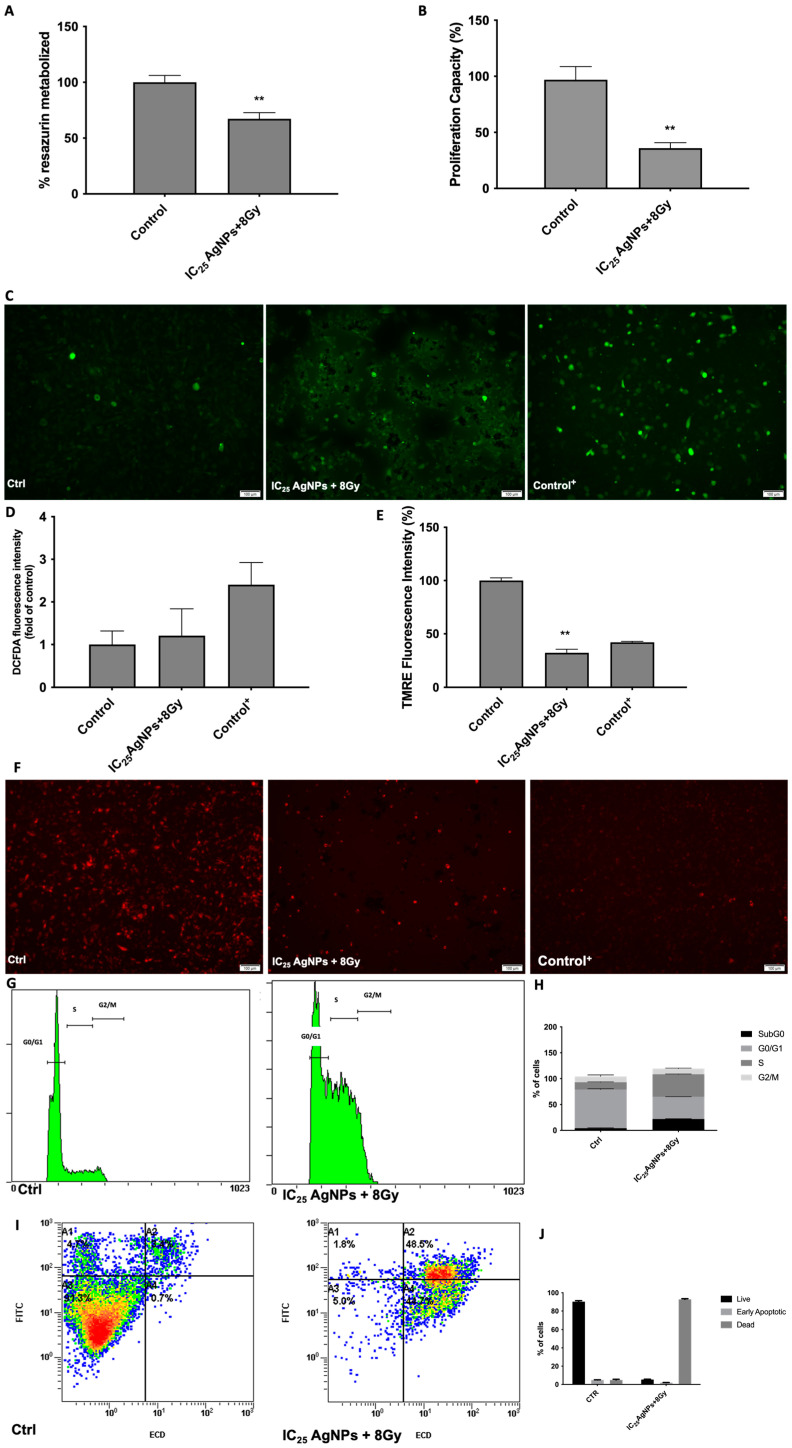
Evaluation of the phenotypic effect of 8 Gy Radiation after exposure to IC_25_ AgNPs for 24 h in 786-O cells. (**A**) Cell viability assessment of cells exposed to 8 Gy radiation without and with the previous administration of IC_25_ AgNPs by Resazurin Assay. Results are expressed as a percentage of control (untreated cells), as mean ± SEM. Statistical analysis was performed in comparison to control; ** *p* < 0.001. (**B**) Proliferation capacity assessment of cells exposed to 8 Gy radiation without and with previous administration of IC_25_ AgNPs by BrDu Assay. Results are expressed as a percentage of control (untreated cells), as mean ± SEM. Statistical analysis was performed in comparison to control; ** *p* < 0.001. (**C**) Evaluation of ROS production of cells exposed to 8 Gy radiation without and with the previous administration of IC25 AgNPs by DCFH2-DA assay; Representative Images (10×) taken using an Olympus IX51 microscope. (**D**) Graph representing the intensity of the DCFH2-DA fluorescence signal. T-bhp represents positive control. Results are expressed as fold of control (untreated cells), as mean ± SEM Statistical analysis was performed in comparison to control; (**E**) Graph representing the intensity of the TMRE fluorescence signal. FCCP represents positive control. Results are expressed as a percentage of control (untreated cells), as mean ± SEM. Statistical analysis was performed in comparison to control; ** *p* < 0.001. (**F**) Evaluation of mitochondria membrane depolarization of cells exposed to 8 Gy radiation without and with the previous administration of IC_25_ AgNPs, by TMRE Assay; Representative Images (10×) taken using an Olympus IX51 microscope. (**G**) Representative DNA histogram of cell cycle analysis of cells exposed to 8 Gy radiation without and with the previous administration of IC_25_ AgNPs, evaluated through PI stain and flow cytometry. (**H**) Descriptive analysis of the number of cells in each phase of the cell cycle. Data are expressed as mean ± SEM. (**I**) Representative flow cytometry images of annexin (FITC) and PI (ECD) staining regarding apoptosis assessment of exposure to 8 Gy radiation without and with previous administration of IC_25_ AgNPs. The different colors in the picture represent the crescent density of cells (blue-less density; red-more density). (**J**) Descriptive analysis of the number of live, early apoptotic and dead cells; Data are expressed as mean ± SEM.

Confirming this resistance, we proceeded to the evaluation of AgNPs’ capacity to sensitize 786-O cells to radiation. To do so, we administrated the IC_25_ AgNPs to 786-O cells. After 24 h, dead cells were removed by PBS washing, and the remaining were exposed to 8 Gy radiation. Then, 1 h after exposure, cells’ viability, proliferation capacity, ROS production, mitochondrial membrane polarization, cell cycle status and apoptosis were evaluated (Figure 7).

Interestingly, there was a reduction of cell viability to 67.37% in cells exposed to 8 Gy after being sensitized with IC_25_ of AgNPs (*p* < 0.001) (Figure 7A). This was followed by a reduction of proliferation capacity to 35.89% (*p* < 0.001) (Figure 7B). When analyzing mitochondrial function (Figure 7C,F), no increase in ROS production was observed (*p* = 0.633) (Figure 7D); however, there was a significative decrease of polarized mitochondrial membrane to 32.13% (*p* < 0.001) (Figure 7E). Regarding the cell cycle apoptotic status of cells when exposed to radiation after being sensitized with IC_25_ of AgNPs, disruption can be observed (Figure 7G,I). There was a decrease in the number of cells in the G0/G1 phase (74.76% in control cells vs. 42.94% in cells sensitized by AgNPs and submitted to radiation) and an increase in the number of cells in the S phase (13.93% in control cells vs. 43.44% in cells sensitized by AgNPs and submitted to radiation). Additionally, there was also an increase in the number of cells in subG0 (4.31% in control cells vs. 22.06% in cells sensitized by AgNPs and submitted to radiation). (Figure 7H). Regarding their apoptotic state, one can see that cells were marked positive for both Annexin and PI (thereby, being apoptotic) only when radiation followed the sensitization with IC_25_ of AgNPs (Figure 7J).

## 4. Discussion

One of the main concerns in ccRCC is the metastatic disease patients’ management since the 5-year survival rate of these patients is lower than 10% [13]. Therefore, the definition of new agents that can effectively treat or enhance and sensitize current therapies is a necessity in this tumor model. The advent of nanomedicine opened the doors to the definition of several potential new therapeutic agents, promising to revolutionize different biomedical fields such as oncology [14]. AgNPs arose attention due to their antitumor effect and easy and cheap synthesis [15]. In fact, they have been widely tested in different tumor models with promising effects and good tolerability [9]. However, very limited research has been performed regarding AgNPs’ effect in ccRCC and, to the best of our knowledge, no author has attempted to test AgNPs’ potential as enhancers or sensitizers of current therapies [16,17].

In the present study, we observed a dose-dependent cytotoxic effect in the 786-O cell line but not in RCC-FG2 and Caki-1. It is known that AgNPs show heterogeneous effects according to the cell line, with their effect being cell type-specific, and the lower effect in these cells may be explained by their more aggressive phenotype [18]. Interestingly, the mechanism of action in 786-O is consistent with what our group previously observed [10]. This mechanism is common to many studies with other AgNPs where authors report an increase in ROS production, mitochondrial damage, nuclear fragmentation and cell apoptosis [9]. In this case, as shown by TEM analysis, AgNPs enter cells through endocytosis and are trapped in lysosomes, which have hydrolytic enzymes that can decompose the capping and release Ag^+^ [19]. These ions will induce the production of ROS, increasing oxidative damage in the different organelles. Interestingly, in the present study, the increase in ROS induced by AgNPs was even higher than the signal induced by t-bhp (the positive control of the experiment), suggesting that ROS production is indeed a key player in AgNP’s toxicity. In this study, we observed significant damage in mitochondria and a blockage of the cell cycle in the S phase. The S Phase checkpoint is responsible for DNA damage check, which can indicate that AgNPs also induce damage in the nucleus, supporting the cell arrest observed in the present study [20]. Moreover, this damage is enough to trigger apoptosis, as observed with FITC annexin V and PI staining. Additionally, the results obtained through the FITC annexin V and PI staining assay, strengthen the results observed through resazurin assay, since they can be a marker of the percentage of viable cells in opposition to the ones in apoptosis and necrosis.

Additionally, we studied AgNPs’ cytotoxic capacity against HepG-2. HepG-2 is a hepatoma cell line widely used to study liver function and hepatoxicity, mostly because they retain several features of differentiated hepatocytes, including insulin-stimulated glycogen synthesis, glutathione-based detoxification and albumin secretion [21]. Since the liver is the site of the first step of metabolism, it is vulnerable to nanoparticle toxicity, making the evaluation of hepatocellular toxicity of uttermost significance [22]. In the present study, we observed a viability of about 82% after administration of AgNPs at the IC_50_ of 786-O, showing that HepG-2 seems less sensitive to AgNPs compared with ccRCC cells. Even though this is a good first indicator of biocompatibility, further studies should be performed regarding lung, dermal and neurotoxicity as well as in vivo studies to ensure AgNPs’ safety.

Considering the demonstrated cytotoxic potential of AgNPs in 786-O cells, we questioned if a low dose of AgNPs would be able to weaken the cells and potentiate the targeted therapy everolimus or sensitize them to radiotherapy. Indeed, the combination of everolimus and AgNPs is of special interest since they seem to share a common target: mTOR. Indeed, the mechanism of action of everolimus is characterized by an inhibition of proliferation through mTOR targeting, and several studies have reported that AgNPs’ administration to HT22, A549, Caco-2, HC11 and HEK293T cells lead to an inhibition of mTOR and phosphor-mTOR and consequent induction of autophagy or apoptosis [11,23,24,25,26]. Added to this potential common target, it is important to notice that AgNPs also induce mitochondrial damage. Therefore, the mTOR inhibition combined with mitochondrial damage seems to be responsible for the enhanced disruption of cell cycle, blockage of proliferation and cell death that we observed in the present study. These results come in accordance with the rising interest in combination therapy in the oncology field since the synergic or additive effect of two drugs combined leads to a need for a lower therapeutic dosage of each individual drug [27]. Some authors have already reported the usage of Everolimus, alone or in combination with other drugs, encapsulated by nanoparticles with promising results but, to the best of our knowledge, there are still no studies reporting the combined effect of AgNPs and Everolimus [28,29,30].

Regarding the potential of AgNPs to sensitize 786-O cells to radiation, one can observe that 786-O cells seem to show intrinsic resistance to radiation as high as 20 Gy, making them a good model to study radiotherapy resistance in ccRCC. Intrinsic resistance to radiation of cell lines has already been reported in different tumor models due to different gene expression profiles [31]. For instance, high expression of *HIF-1α* has been shown to be correlated with resistance to radiation on human oral squamous cell carcinoma cell lines [32]. The 786-O cell line has a mutated *VHL*, with a defective VHL expression and, consequent, high expression of HIF-1α, which may explain the observed resistance to radiation [18]. Interestingly, after administration of AgNPs, the surviving cells were sensitized to radiation. To the best of our knowledge, no other study has reported a sensitizing effect of AgNPs to radiation in ccRCC, but different authors have already shown AgNPs’ potential to enhance the radiotherapy effect in different tumor models [33,34]. In fact, previous studies have reported that the damage induced in mitochondria by AgNPs can trigger a variety of mitochondrial apoptotic stimulators that are released to the cytoplasm and can turn cells more prone to apoptosis, therefore sensitizing them to radiation [34]. In our study, we observed that, after AgNPs administration, radiation led to a disruption of the cell cycle, which blocked proliferation and triggered apoptosis. Radiation-induced DNA damage is one of the key triggers for activation of DNA structure checkpoints leading to cell cycle arrest [35]. We observed a cell cycle arrest at the S-phase. In fact, different damages in DNA, such as double-strand breaks (one of the most common damage induced by radiation), are known to trigger the arrest of the cell cycle in the S-phase [36]. Therefore, we hypothesize that the mitochondrial damage induced by AgNPs will release apoptotic stimulators that will make cells more prone to radiation. Consequently, radiation will cause DNA damage, probably double-strand breaks, that will cause an arrest in the S-phase and, altogether, lead to apoptosis.

It is important to notice that targeted therapies and radiotherapy are mostly used in the metastatic context of ccRCC. However, one should take into consideration that up to 17% of ccRCC patients harbor distant metastasis at the time of the diagnosis and up to 40% of patients submitted to surgery with curative intent relapse in a 5-year period [2,37]. Therefore, the question of whether, if identifying these patients, they would benefit better from a more radical approach using targeted or radiotherapy in an earlier onset arises. In the present study, we show that a combination of AgNPs and everolimus or radiotherapy could be an effective approach in these scenarios. Nevertheless, it would be important in the future to study the effect of these combinatory schemes in metastatic cell lines to understand if the results are replicated.

## 5. Conclusions

In this study, we demonstrated the potential of AgNPs administrated in combination with current therapeutic options to treat ccRCC. Due to the rising populational, social and economic burden of cancer, it is important to develop therapeutic strategies that are safe, efficient and cost-effective. Thus, the ability to lower therapeutic doses or sensitize to current therapies is a major advantage of new therapeutic agents. There are still very few studies regarding AgNPs and ccRCC, and, to the best of our knowledge, none has explored their combinatory effect with Everolimus and radiotherapy. The AgNPs tested in this study share some of the characteristics mentioned above and were able to enhance the effects of Everolimus and sensitize cells to radiotherapy. However, this is an initial study and needs further validation in vivo to understand the penetration ability of AgNPs and confirm their cytotoxic potential and excretion pathway safety. Additionally, it would be important to understand if the side effects commonly described for Everolimus in vivo can be reduced using low doses of AgNPs and this drug. Moreover, regarding radiotherapy, it would be interesting to understand which pathways are involved in this sensitization after AgNPs administration. Nevertheless, this first study displays promising results and shows that these AgNPs may be part of potential therapeutic strategies that can help improve the management of ccRCC patients.

## Figures and Tables

**Table 1:** IC_25_ and IC_50_ of AgNPs in 786-O cell line.

IC_25_	IC_50_
1712.15 µg/mL	2629.63 µg/mL

## Data Availability

Data available on request.
